# Placental expression of imprinted genes varies with sampling site and mode of delivery

**DOI:** 10.1016/j.placenta.2015.06.011

**Published:** 2015-08

**Authors:** A.B. Janssen, S.J. Tunster, N. Savory, A. Holmes, J. Beasley, S.A.R. Parveen, R.J.A. Penketh, R.M. John

**Affiliations:** a Cardiff School of Biosciences, Cardiff University, Cardiff, Wales, CF10 3AX, UK; b Department of Obstetrics and Gynaecology, University Hospital Wales, Cardiff, Wales CF144XW, UK; c Department of Obstetrics and Gynaecology, Royal Gwent Hospital, Newport, Wales NP202UB, UK

**Keywords:** Placenta, Imprinted genes, Sampling, Delivery mode

## Abstract

Imprinted genes, which are monoallelically expressed by virtue of an epigenetic process initiated in the germline, are known to play key roles in regulating fetal growth and placental development. Numerous studies are investigating the expression of these imprinted genes in the human placenta in relation to common complications of pregnancy such as fetal growth restriction and preeclampsia. This study aimed to determine whether placental sampling protocols or other factors such as fetal sex, gestational age and mode of delivery may influence the expression of imprinted genes predicted to regulate placental signalling.

**Methods:**

Term placentas were collected from Caucasian women delivering at University Hospital of Wales or Royal Gwent Hospital within two hours of delivery. Expression of the imprinted genes *PHLDA2*, *CDKN1C*, *PEG3* and *PEG10* was assayed by quantitative real time PCR. Intraplacental gene expression was analysed (N = 5). Placental gene expression was compared between male (N = 11) and female (N = 11) infants, early term (N = 8) and late term (N = 10) deliveries and between labouring (N = 13) and non-labouring (N = 21) participants.

**Results:**

The paternally expressed imprinted genes *PEG3* and *PEG10* were resilient to differences in sampling site, fetal sex, term gestational age and mode of delivery. The maternally expressed imprinted gene *CDKN1C* was elevated over 2-fold (p < 0.001) in placenta from labouring deliveries compared with elective caesarean sections. In addition, the maternally expressed imprinted gene *PHLDA2* was elevated by 1.8 fold (p = 0.01) in samples taken at the distal edge of the placenta compared to the cord insertion site.

**Conclusion:**

These findings support the reinterpretation of existing data sets on these genes in relation to complications of pregnancy and further reinforce the importance of optimising and unifying placental collection protocols for future studies.

## Highlights

• 77% variation in expression of *PHLDA2* between distal and proximal sampling sites.
• 2.4-fold increase in placental expression of *CDKN1C* induced by labour.
• May explain conflicting links between pregnancy pathologies and imprinted genes.

## 1 Introduction

Imprinted genes display parent of origin specific gene expression [1] and play a key role in regulating placental development and controlling fetal growth (reviewed in Ref. [2]). Recent studies have identified several imprinted genes that regulate the endocrine lineages of the mouse placenta to modulate placental hormone production (reviewed in Ref. [3]). Imprinting of four of these genes, *PLECKSTRIN HOMOLOGY-LIKE DOMAIN FAMILY A MEMBER 2* (*PHLDA2*), *CYCLIN-DEPENDENT KINASE INHIBITOR 1C* (*CDKN1C*), *PATERNALLY EXPRESSED GENE 3* (*PEG3*) and *PATERNALLY EXPRESSED GENE 10* (*PEG10*), is conserved in the human placenta [4]. While a number of studies have examined human placental imprinted gene expression focusing primarily on fetal growth restriction (FGR) as an adverse outcome [4–9], the potential for aberrant placental signalling presents additional implications for human pregnancies.

Gene expression in the placenta can be influenced by many factors including differences in sampling site, fetal sex, gestational age and mode of delivery (reviewed in Ref. [10]). Variation in gene expression may occur in different regions of the placenta due to differences in placental architecture or blood supply and therefore consistency in placental sampling is of key importance [10]. A number of studies have demonstrated differences in intraplacental gene expression depending on sampling site [11–17] but the possibility of intraplacental variation in expression of *PHLDA2*, *CDKN1C*, *PEG3* or *PEG10* has not been investigated.

Sexual dimorphism in human placental gene expression (of both autosomal and sex chromosome linked genes) has also previously been demonstrated [17,18] which has been proposed to underlie sex differences in fetal growth [19]. As sexual dimorphism in placental imprinted gene expression has been demonstrated in an animal model [20] and for the imprinted *H19* gene in human placenta [9], controlling for fetal sex is important to studies of imprinted gene expression in the human placenta.

Infant morbidity and mortality [21–23] as well as placental pathology [24] have been observed to vary in response to gestational age in term pregnancies. It has therefore been proposed that infants should be distinguished as early term (37 0/7 to 38 6/7 weeks) and full term (39 0/7 to 41 6/7) [23]. While it has been demonstrated that placental expression of *PHLDA2* varies between preterm and term deliveries [7], it has not yet been established whether expression of *PHLDA2*, *CDKN1C*, *PEG3* or *PEG10* varies between early term and full term placentas. This is of particular relevance to the study of pregnancy complications such as FGR, as these growth restricted infants may require earlier delivery [25].

Importantly, a number of studies have reported differences in gene expression between labouring and non-labouring placentas [26–29], which may arise from a decrease in placental oxygen supply during contractions [10] and/or differences in exposure to hormones associated with labour [30]. *PHLDA2* is known to be responsive to hypoxia [31–33] but it is not known whether placental expression of *PHLDA2*, *CDKN1C*, *PEG3* or *PEG10* is altered in response to labour.

To address this lack of information, this study examined placental expression of the maternally expressed imprinted genes *PHLDA2* and *CDKN1C* and the paternally expressed imprinted genes *PEG3* and *PEG10* to determine whether their expression varied in relation to sampling site, fetal sex, gestational age or mode of delivery.

## 2 Methods

### 2.1 Study participants

Study participants (N = 40) were recruited prior to delivery at University Hospital Wales, Cardiff and Royal Gwent Hospital, Newport. Written informed consent was obtained and the study was approved by the South East Wales Research Ethics Committee (REC number: 10/WSE02/10). Healthy Caucasian women with singleton pregnancies and no known medical disorders were recruited and all infants were delivered normal birth weight at term (≥37 weeks). Information on birth outcomes were obtained from the maternal medical notes.

In order to determine the effect of fetal sex on placental imprinted gene expression, gene expression was compared between male (N = 11) and female (N = 11) placentas delivered by elective C-section. Gene expression was also compared between early term (N = 8) and full term (N = 10) placentas delivered by elective C-section to determine whether expression varies with gestational age at term. Finally, to determine whether labour effects placental imprinted gene expression, gene expression was compared between placentas delivered by elective C-section in the absence of labour (N = 21) and by vaginal (N = 9) or emergency C-section delivery following a period of labour (N = 4). Allocation of participants to each group and participant characteristics is summarized in Supplementary Table 1.

### 2.2 Placental dissection

Placentas were weighed and dissected within 2 h of delivery. Villous trophoblast samples were obtained at five random sites on the maternal surface of the placenta, midway between the cord and distal edge. Samples were thoroughly rinsed in ice cold phosphate buffered saline to remove excess blood. Placental samples were then stored in RNA*later* (Sigma–Aldrich, Dorset, UK) at 4 °C prior to long term storage at −80 °C.

To examine intraplacental variation in imprinted gene expression, three placental samples (close to the cord insertion, middle and distal edge) were taken from each of the fetal, middle and maternal layers of the placenta as described by Wyatt et al. (2005) (16). This was carried out for five term placentas (2 male, 3 female) from elective C-sections. Average placental gene expression (fetal, middle and maternal layers of the placenta) was compared between sampling sites close to the cord insertion and at distal sites. Similarly, average placental gene expression (cord insertion, middle and distal edge) was compared between basal and chorionic surface sampling sites.

### 2.3 Gene expression analysis

Total RNA was extracted from the placental tissue samples using GenElute Mammalian Total RNA Miniprep Kit (Sigma–Aldrich, Dorset, UK) with an on-column DNase digestion (Sigma–Aldrich, Dorset, UK). 5 μg of RNA was reverse transcribed using M-MuLV reverse transcriptase (Promega, Southampton, UK) with random hexamers, according to manufacturer's instructions.

Quantitative RT-PCR was performed using Chromo 4 Four Colour Real Time Detector (MJ Research) in a 20 μl reaction containing 5 μl of cDNA (diluted 1 in 50), 1X Buffer 2 mM MgCl_2_, 2 mM dNTPs, 0.65 Units Taq (Fermentas (Thermo), Loughborough, UK), 1 μM of each primer (Sigma–Aldrich, Dorset, UK) and 0.12X Sybr Green (Invitrogen, Paisley, UK). All samples were run in triplicate and duplicate plates were performed. Conditions for amplification were: 1) 15 min at 94 °C, 2) 30 s at 94 °C, 3) 30 s at 60 °C, 4) 30 s at 72 °C and 5) 30 s 75 °C, with steps 2–5 repeated for a total of 40 cycles. Melt Curve was performed from 70 °C to 94 °C, reading every 0.5 °C and holding for 2 s.

Primer sequences were as follows: *YWHAZ* forward: TTCTTGATCCCCAATGCTTC and reverse: AGTTAAGGGCCAGACCCAGT, *PHLDA2* forward: GAGCGCACGGGCAAGTA and reverse: CAGCGGAAGTCGATCTCCTT [6], *CDKN1C* forward: CCCATCTAGCTTGCAGTCTCTT and reverse: CAGACGGCTCAGGAACCATT [4], *PEG3* forward: CTCACAACACAATCCAGGAC and reverse: TAGACCTCGACTGGTGCTTG, *PEG10* forward: AAATTGCCTGACATGAAGAGGAGTCTA and reverse: AAGCCTAGTCACCACTTCAAAACACACTAAA [4].

Gene expression data is presented as the ΔCT (target gene expression relative to the housekeeping gene *YWHAZ*) and as the fold change in expression, calculated using the 2^−ΔΔCT^ [34] where the ΔΔCT is the target gene expression relative to expression in the control group. The housekeeping gene *YWHAZ* was chosen as this gene has been demonstrated in a number of studies to be stably expressed in the human placenta of normal pregnancies [35–37] and in pregnancies complicated by growth restriction [38]. In the current study there was no significant effect of fetal sex (p = 0.56), early term delivery (p = 0.44) or labour (p = 0.74) on placental *YWHAZ* gene expression.

### 2.4 Statistical analysis

All statistical analysis was carried out using IBM SPSS Statistics for Windows (version 20.0, 2011). A Shapiro–Wilk test was used to test for normal distribution of the data. All gene expression data was normally distributed and therefore differences in gene expression were analysed using an independent samples T-test.

## 3 Results

A summary of participant demographics is shown in Table 1.

### 3.1 Sampling site variation

**Fig. 1:**
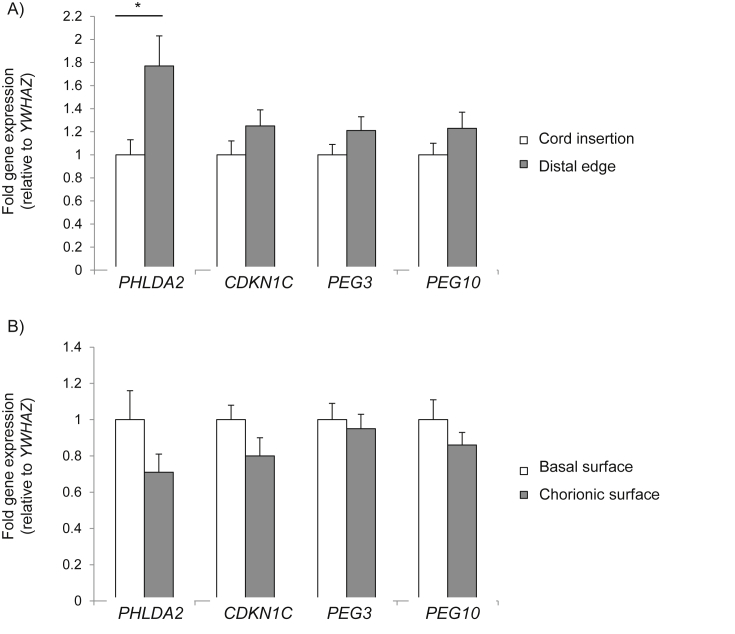
**Effect of sampling site on placental imprinted gene expression**. Gene expression is shown relative to sampling site near the cord insertion (A) and on the basal surface (B) of the placenta. Mean fold gene expression is shown (±SEM). Three samples were taken at each site for a total of five placentas (N = 15 samples per site). Differences in gene expression were analysed using an independent samples t-test. *p < 0.05.

Placental *CDKN1C*, *PEG3* and *PEG10* expression did not differ significantly between cord insertion and distal sampling sites (Fig. 1A) or between the basal and chorionic surface (Fig. 1B). Placental *PHLDA2* expression was significantly increased, by 77%, in samples taken at distal sampling sites compared with sampling sites close to the cord insertion (Fig. 1A). There was no significant difference in *PHLDA2* expression between sampling sites on the basal and chorionic placental surface (Fig. 1B).

### 3.2 Fetal sex

**Fig. 2:**
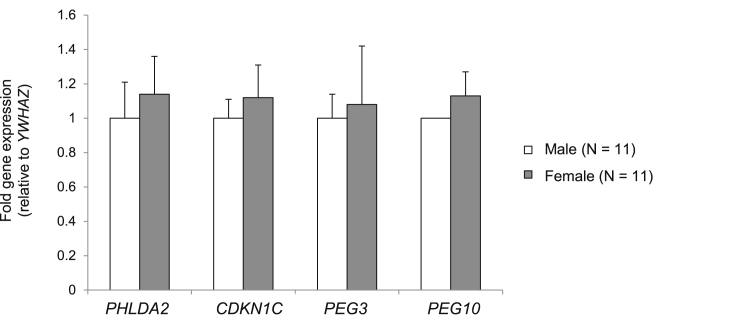
**Sex differences in placental imprinted gene expression**. Mean fold gene expression is shown (±SEM) relative to expression in male placentas. Differences in gene expression were not statistically significant using an independent samples t-test.

There was no significant difference in mean placental weight (663 g vs. 726 g; *p =* 0.23), birth weight (3381 g vs. 3601 g; p = 0.07) or gestational age (273 days v 274 days; p = 0.85) between male and female infants. There was no significant difference in placental *PHLDA2*, *CDKN1C*, *PEG3* or *PEG10* expression between male and female placentas (Fig. 2).

### 3.3 Gestational age

**Fig. 3:**
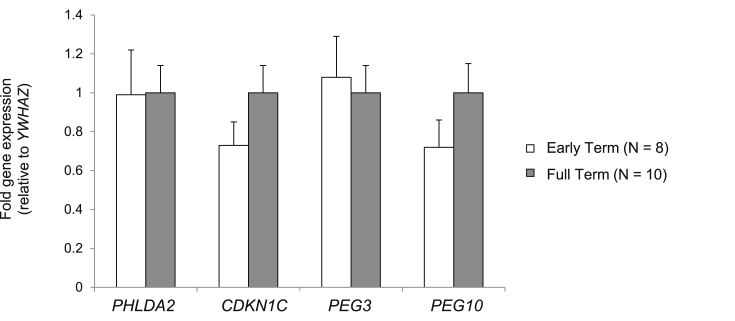
**Early term delivery and placental imprinted gene expression**. Mean fold gene expression is shown (±SEM) relative to expression in full term placentas. Differences in gene expression were not statistically significant using an independent samples T-test.

Early term infants were delivered between 37 and 38 weeks (mean gestational age 266 days) and full term infants between 39 and 40 weeks (mean gestational age 277 days). There was no significant difference in mean placental weight (648 g vs. 717 g; p = 0.23), birth weight (3445 g vs. 3392 g; p = 0.07) between early term and full term infants. The proportion of male and female infants was also not significantly different between early (4 Male, 4 Female) and full term (4 Male and 6 Female) participants (p = 0.67). *PHLDA2*, *CDKN1C*, *PEG3* and *PEG10* expression was not significantly altered in early term compared with full term placentas (Fig. 3).

### 3.4 Labour

There was no significant difference in mean placental weight (708 g vs. 661 g; p = 0.31), birth weight (3470 g vs. 3451 g; p = 0.85) or gestational age (273 days v 276 days; p = 0.49) between labouring and non-labouring participants. There was also no significant difference in the proportion of male and female infants between labouring (6 Male, 7 Female) and non-labouring (11 Male, 10 Female) participants (p = 0.72).

**Fig. 4:**
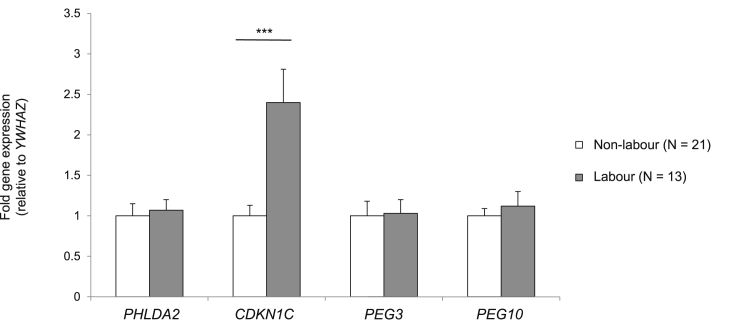
**Effect of labour on placental imprinted gene expression**. Mean fold gene expression is shown (±SEM) relative to expression in placentas from elective C section deliveries. Differences in gene expression were analysed using an independent samples T-test. ***p < 0.001.

There was no significant effect of labour on placental *PHLDA2*, *PEG3* or *PEG10* expression (Fig. 4). Placental *CDKN1C* was increased, by 140%, in labouring compared with non-labouring participants (Fig. 4). This increase in placental *CDKN1C* expression remained significant when labouring participants were divided into those delivering vaginally (115% increase, p = 0.001, n = 9) and those delivering by emergency C-section (197% increase, p = 0.001, n = 4).

## 4 Discussion

This study demonstrates that both the site of sampling and the mode of delivery can influence the quantitative assessment of imprinted gene expression in the human placenta.

Our first key finding was that the expression of *PHLDA2* varied with site of sampling. *PHLDA2* is a maternally expressed imprinted gene whose elevated placental expression has been reported in relation to fetal growth restriction in several human studies [39]. In mice we have shown that transgenically elevated *Phlda2* drives a late and asymmetric fetal growth restriction [40] suggesting a causal role for *PHLDA2* in human fetal growth restriction. In this current study, we found that placental *PHLDA2* expression differed significantly with sampling site, with increased expression at distal sampling sites compared with sites close to the umbilical cord. Although no previous study has examined placental *PHLDA2* expression in relation to sampling site, a number of studies have similarly reported significant intraplacental variation in gene expression [12–14,16,17]. Given that placental *PHLDA2* expression is altered in response to hypoxia [31–33], it is possible that differences in perfusion between distal sites and sites close to the umbilical cord [14,16] could underlie the differences in expression observed. The finding of intraplacental variation in *PHLDA2* expression is novel and has implications for interpretation of previous studies and for future studies with consistency in placental sampling of key importance.

A second key finding in this study was the variation in *CDKN1C* in relation to mode of delivery. In humans, loss of expression of *CDKN1C* has been linked to the childhood overgrowth disorder Beckwith Weidemann Syndrome while microduplications spanning the gene and alterations in the PCNA domain of *CDKN1C* have been linked to the growth restriction disorders Silver-Russell syndrome (SRS) and IMAGe syndrome, respectively [41]. Mouse studies demonstrate that *Cdkn1c* functions early in life to limit fetal growth and to regulate placental development [42–45] consistent with a role for this gene in these disorders.

Loss of function of *CDKN1C* may also play a role in preeclampsia/HELLP syndrome [46] which has also been partially supported by animal studies [47]. However, while one study reported significantly reduced *CDKN1C* expression in preeclamptic placentas [48] two other studies reported significantly elevated expression [49,50]. In the study by Kawasaki et al. [48], placental villous samples were obtained immediately after caesarean section in the absence of labour for both controls and preeclampsia pregnancies that would exclude mode of delivery as a confounder. In the study by Enquobahrie et al. [50] preeclamptic pregnancies had a higher caesarean delivery rate but a similar rate of labour with controls while in the study by Unek et al. [49] placenta were sampled after caesarean delivery without stating whether labour was initiated. Similarly, studies examining placental *CDKN1C* expression in relation to fetal growth restriction have reported conflicting results with both unaltered [4], increased [5] and decreased [51] expression reported in FGR placentas which may result from a failure to control for mode of delivery in these analyses.

In this study, the expression of *CDKN1C* varied in response to mode of delivery with both vaginally delivered and emergency caesarean placenta expressing *CDKN1C* at significantly higher levels than placenta obtained by elective caesarean in which labour was not initiated. This is consistent with previous reports of significantly increased *CDKN1C* expression in the mouse uterus and human myometrium in association with labour [52]. In light of these new findings, it will be important to re-examine the data both from studies reporting a link between placental *CDKN1C* and preeclampsia or fetal growth restriction and those studies finding no association.

No significant difference was observed in placental *PHLDA2*, *PEG3* or *PEG10* expression in response to labour, and no intraplacental variation was observed in placental *CDKN1C*, *PEG3* or *PEG10* expression at different sampling sites. None of the four genes showed a significant difference in expression between early and late term deliveries and there was no significant difference in relation to fetal sex confirming the findings of Moore et al. [9]. However, sexual dimorphism exists in the human fetal response to an adverse environment (reviewed in Ref. [19]) and differential imprinted gene expression has been reported in male and female mouse placentas in response to maternal diet alteration [53]. Thus, a sex-specific response in human placental imprinted gene expression to an adverse intrauterine environment merits further investigation.

In conclusion, we have found that the expression of *PHLDA2* varied with sampling site and that expression of *CDKN1C* varied with mode of delivery. These types of study further highlight the importance of developing uniform collection protocols with details on mode of delivery as well as birth outcomes [10].

## Figures and Tables

**Table 1:** **Characteristics of the study participants (N** = **40)**. Mean (SD)/range or number (%) is shown.

	Study Participants (N = 40)
***Maternal Characteristic***	
Ethnicity:	
Caucasian	40 (100%)
Parity	1 (0.87)/0–3
Maternal age	30 (6.12)/19–40
Maternal BMI	27 (5.91)/17 - 38
***Birth Outcome***	
Mode of Delivery:	
*Vaginal*	9 (22%)
*Elective C section*	27 (68%)
*Emergency C section*	4 (10%)
Birth weight (g)	3417 (303)/2680 - 4030
Gestational age (weeks)	39 (1.19)/37 - 42
Placental weight (g)	676 (120)/434 - 891
Gender	
*Male*	18 (45%)
*Female*	22 (55%)
