# Supplementary material for: Placental expression of imprinted genes varies with sampling site and mode of delivery
Source: Placenta. 2015 Aug;36(8):790–5. doi: 10.1016/j.placenta.2015.06.011 (PMC4535278; doi:10.1016/j.placenta.2015.06.011)
Supplement: Supplementary file 1 [file mmc1.docx]

**Supplementary Table 1**: Participant characteristics for individual comparison groups. ELCS = elective caesarean section, EMCS = emergency caesarean section. Number and (%) shown.

|  | **Fetal Sex** | **Gestational age** | **Mode of delivery** |
| --- | --- | --- | --- |
| **Sampling Site analysis** (n=5) | Male: 2 (40%)  Female: 3 (60%) | Early Term: 1 (20%)  Full Term 4 (80%) | ELCS: 5 (100%)  EMCS: 0 (0%)  Vaginal: 0 (0%) |
| **Sex differences analysis** (n= 22) | Male: 11 (50%)  Female: 11 (50%) | Early Term: 10 (45%)  Full Term: 12 (55%) | ELCS: 22 (100%)  EMCS: 0 (0%)  Vaginal: 0 (0%) |
| **Gestational age analysis** (n = 18) | Male: 8 (44%)  Female: 10 (56%) | Early Term: 8 (44%)  Full Term 10 (56%) | ELCS: 18 (100%)  EMCS: (0%)  Vaginal: (0%) |
| **Labour analysis**  (n = 34) | Male: 17 (50%)  Female: 17 (50%) | Early Term: 15 (44%)  Full Term 19 (56%) | ELCS: 21 (62%)  EMCS: 4 (12%)  Vaginal: 9 (26%) |
